# Family doctors' involvement with families in Estonia

**DOI:** 10.1186/1471-2296-5-24

**Published:** 2004-10-25

**Authors:** Marje Oona, Ruth Kalda, Margus Lember, Heidi-Ingrid Maaroos

**Affiliations:** 1 Dept of Polyclinic and Family Medicine, University of Tartu, Puusepa 1a, 50406 Tartu, Estonia; 2 Dept of Internal Medicine, University of Tartu, Puusepa 6, 51014 Tartu, Estonia

## Abstract

**Background:**

Family doctors should care for individuals in the context of their family. Family has a powerful influence on health and illness and family interventions have been shown to improve health outcomes for a variety of health problems. The aim of the study was to investigate the Estonian family doctors' (FD) attitudes to the patients' family-related issues in their work: to explore the degree of FDs involvement in family matters, their preparedness for management of family-related issues and their self-assessment of the ability to manage different family-related problems.

**Methods:**

A random sample (n = 236) of all FDs in Estonia was investigated using a postal questionnaire. Altogether 151 FDs responded to the questionnaire (response rate 64%), while five of them were excluded as they did not actually work as FDs.

**Results:**

Of the respondents, 90% thought that in managing the health problems of patients FDs should communicate and cooperate with family members. Although most of the family doctors agreed that modifying of the health damaging risk factors (smoking, alcohol and drug abuse) of their patients and families is their task, one third of them felt that dealing with these problems is ineffective, or perceived themselves as poorly prepared or having too little time for such activities. Of the respondents, 58% (n = 83) were of the opinion that they could modify also relationship problems.

**Conclusions:**

Estonian family doctors are favourably disposed to involvement in family-related problems, however, they need some additional training, especially in the field of relationship management.

## Background

There are significant differences in the way how primary health care is organised in Europe [1]. Estonia was one of the first Eastern European countries where modern general practice was implemented [2]. Previously, the primary health care system functioned according to the Soviet model, which was basically a specialist-oriented system [3]. In the 1990s, there occurred a transition to a more personal, comprehensive continuous care on the primary level. In 1991, the training of family doctors was launched, both 3-year postgraduate residence training, as well as the retraining of currently practising primary care physicians through attending different courses at the University of Tartu parallel with their everyday practice. The courses on family practice covered such topics as special features of family practice, common clinical problems in family practice, diagnostic strategies, teamwork, ethical issues, prevention and health promotion [3]. For the population, the most important change was the introduction of the patient's list system for FD's: persons choose their own FD by registering in a patient list. Currently, all primary health care physicians in Estonia are trained family doctors who are able to provide a wide scope of medical services for their patients and fulfilling gate-keeping function for specialized medical care.

Irrespective of the health system, general practitioners/family doctors should care for individuals in the context of their family [1]. Family has a powerful influence on health and illness and family interventions have been shown to improve health outcomes for a variety of health problems [4]. However, there are differences in the physicians' involvement with families. Doherty and Baird have described five levels of physician involvement with families [5]. In Estonia, it has been aimed that a family doctor should work at least at level three, i.e. he or she has to communicate appropriate medical information and advice to family members, to be aware of gross family dysfunctions and to deal with the family members' feelings and concerns related to the condition of the patient [5,6]. It may differ in different countries what is expected and valued in general practice care [7]. What constitutes good medical care is determined culturally within a specific historical and geographic context [8].

In Estonia, a decade has passed after the new speciality, family doctor, was introduced into the health care system. The aim of the present study was to investigate the attitude of FDs in Estonia to family-oriented general practice [9]: FDs' awareness of various family-related matters of their patients, FDs' preparedness for management of family-related issues and FDs' self-assessment of the ability to manage different problems (substance abuse, relationship problems) in the family.

## Methods

A 21-item questionnaire was designed for the study. The items were developed by researchers considering the aims of the study.

First, the FDs were asked whether their patients have registered on the list by families or not and whether they regard it as appropriate that the family should be cared by one doctor, or whether they think that children should have a separate primary care physician. The FDs rated their opinion on the degree of involvement with various problems in the family: they should deal only with the treatment and counselling of a particular patient, or to cooperate also with family members, in addition to treatment of a particular patient, or they should deal also with emotional and relationship problems of the family members. The questions about FDs' awareness of various issues related to their patients' families such as familial diseases and diseases of family members, financial coping, relationships in the family, living conditions (overcrowding), drug addiction, alcohol abuse, smoking and leisure activities were inquired on a three-step scale : yes, in the case of each patient; yes, in certain cases; no, it is not necessary. FDs' were asked to self-estimate their ability to manage problems in families such as substance abuse and relationship problems. Questions about the FDs' ability to manage problems in families were open-ended, for example: how do you assess your possibilities as a FD to influence relationship problems in the family? If you assess that you cannot influence them, please specify why? If you assess you can influence them, please specify how? Also, the FDs were asked to estimate whether their professional training for dealing with the problems of families is adequate or inadequate. Several questions were related to sociodemographic characteristics (sex, age) and professional history (character and size of practices and length of service in primary health care). The questionnaire was piloted for clarity and relevance in a group of five FDs, and minor changes were introduced.

In February 2002, the questionnaire was mailed to 236 FDs. a random sample of family doctors of Estonia. The random sample of FDs was formed by choosing the name of every 3^rd ^doctor, in alphabetical order, from the list of doctors who had passed residency or retraining courses in family medicine by that time in Estonia (n = 715). A note of reminder and a new questionnaire was sent to the non-responders 4 weeks after the first mailing. Of the 236 mailed questionnaires, 151 were returned after two mailings (64%), five of them were excluded as the respondents did not actually work as FDs. Thus, altogether 146 questionnaires were included in the study, of these 124 were fully completed, while in 22 cases some of the answers (1 to 3 per questionnaire) were missing.

The data were analysed using SPSS for the Windows version 10. The chi-square test was used to test the differences in the proportions, all p-values calculated were two-tailed, the p-values higher than 0.05 were considered non-significant (NS).

All open questions were analysed as follows: all statements expressing motivation for or indicating problems with dealing with family issues were marked. Further, all similar expressions were grouped under one category. Proceeding from this, the key problems relevant to the study were identified [10].

## Results

### Respondents' characteristics

The mean age of the respondents was 46 (± 8) years, the mean length of the period during which they had worked in primary health care was 18 (± 9) years and the mean size of the list was 1800 (± 513) patients. Of the respondents 55% worked in urban areas, 40% in rural areas, and 5% worked in both areas. The majority of the doctors (92%) were female. The age and sex distribution of the respondents and non-respondents did not differ significantly.

### Individual versus family registration in patient lists

A total of 90 (62%) FDs were of the opinion that it was good to have the same FD for the whole family, 31 (21%) responded that it was preferable that every family member chooses a FD on the basis of personal preference and 25 (17%) thought that children should have a primary health care physician other than adults of the same family. Of the FDs, 119 (82%) responded that most patients were registered in their lists by families.

### The degree of involvement of FDs in family matters

Of the respondents 15 (10%) were of the opinion that FDs should deal only with the health problems of concrete patients without involvement of family members, 94 (65%) responded that, besides managing the health problems of patients, FDs should communicate and cooperate with family members, and 36 (25%) thought that apart from the previously mentioned issues, FDs should deal with the family members' emotional and relationship problems.

### FDs' belief about the necessity for awareness of different family matters

**Figure 1:**
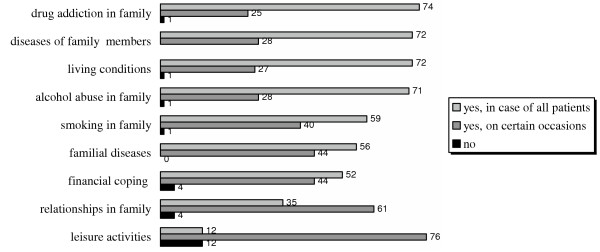
Percentage distribution of the FDs' answers to the question: "Is it necessary to be aware of the following issues related to their patients' families?"

Over 70% of Estonian FDs agreed that in the case of all patients, it is necessary to be aware of drug addiction in the family, diseases of family members, living conditions and alcohol abuse in the family, while the remainder believed that they should be aware of these issues on certain occasions. Of the respondents, over a third thought that the FD should always be aware of relationships in the family and 12% thought that the FD should always be aware of leisure activities of their patients. However, between 60 to 75% believed that FDs should be aware of these issues in certain occasions (Figure 1). Very few FDs responded that patients had never actively sought FDs to discuss family relations (4 out of 143) or health risks in the family (5 out of 145), while 44 (30%) of the respondents stated that patients commonly addressed them to discuss family relations, and 34 (23%) reported that it was common to discuss the health risks associated with the familial diseases.

### Preparedness for management of family-related issues

The respondents valued highly their preparedness to counsel for harmful habits: 104 (71%) of the respondents felt that their preparedness was adequate. Regarding the other issues, less than half of the respondents considered their training adequate (Table 1).

**Table 1:** FDs' self-assessment of their preparedness for management of different family related issues

Issues	Preparedness adequate n (%)	Preparedness inadequate n (%)
Training in counselling for harmful habits	104 (71%)	42 (29%)
Training in counselling for the health risks associated with hereditary diseases	63 (43%)	82 (57%)
Training in relationships counselling	39 (27%)	106 (73%)

p < 0.0001

### FDs' self-assessement of the ability to manage different problems (substance abuse, relationship problems) in family

Altogether 142 FDs responded to the question about their ability to reduce the use of harmful substances (alcohol, tobacco, drugs) in families. One hundred (70%) of the respondents reported that this was within the scope of their ability, while the majority (n = 71) stated that the methods used were advice and counselling, but also referral to specialists, use of specific medications and suggestions regarding appropriate reading material. However, 16 FDs admitted that the efficacy of their work in this field was low.

Nearly one-third of the respondents (30%) estimated that they were not able to reduce the use of harmful substances in their patients' families. The analysis of the open-ended questions identifed some key problems:

• Low motivation of patients.

• Socio-economic reasons for substance abuse.

• Limited time for consultation.

• Inadequate preparedness for management of these issues.

One hundred and forty-three FDs responded to the question about their influence on relationship problems in their patients' families, 83 (58%) of them were of the opinion that they were able to modify these problems. In most cases, FDs used advice and counselling (n = 52), but they also cooperated with specialists as the psychotherapist, family therapist, psychiatrist or social worker. Of the family doctors 60 (42%) thought that they were not able to influence the patients' relationship problems. In the analysis of the open questions, the doctors identified several key problems:

• Limited time.

• Lack of special training.

• Patients do not address FDs with their problem.

• Patients themselves deny the existence of the problem.

• These are the patients' private issues in which physicians could not intervene.

The FDs who were sure that they were able to modify the patients' harmful habits as well as family relationships were more likely to estimate their preparedness for the management of these issues as adequate. Among the doctors who reported that their preparedness for counselling for lifestyle issues was adequate, 76% (n = 76) believed that they were able to treat harmful habits, *versus *57% (n = 24) of those who reported that their preparedness for such issues was not adequate (p < 0.05); in the case of relationship problems, the respective percentages were 41% (n = 34) *versus *9% (n = 5) (p < 0.0001). There were found no other significant determinants among the sociodemographic or work related factors.

## Discussion

The present study addressed the family doctors' opinions about their involvement in the patients' family issues. This is the first study of this kind conducted in Estonia, a country where family doctors were introduced into the health care system ten years ago. There is yet no definite agreement as to what are the appropriate, ideal or minimal levels of family orientation that family doctors should have [9]. Several studies have shown that the frequency of discussing family issues varies significantly [11-13]. The limitation of the study was that the response rate was quite low, 64%. However, the age and sex distribution of the respondents corresponds to that of the Estonian family doctors in general [14].

Our study revealed that care of patients in the context of the family is an important issue for FDs and that Estonian family doctors have good possibilities to take care of the whole family. Although all patients have the right to choose an individual family doctor, the FDs who responded to the questionnaire were sure that family members mostly have one and the same family doctor. This is concordant with the results of a recent survey among patients according to which 74% of the respondents reported that they had one and the same family physician for the whole family [15]. In Estonia, similar with the other Eastern Europe countries shift from separate pediatric and adult primary care system to family doctors system occurred in the 1990s [16]. Only 17% of the FDs in our study were of the opinion that children and adults should have different primary care doctors.

In our study, altogether 90% of the family doctors thought that they should communicate and cooperate with family members in management of the health problems of patients. It is a good result considering that family medicine is a new and developing speciality in Estonia. At the same time, most family doctors are not yet ready to deal with the family members' emotional and relationship problems. Primary care is an important early intervention site of most serious relationship issues, domestic violence, etc [17]. However, studies conducted in other communities have identified that physicians need more continuing education concerning these topics [18,19]. Currently, a two-day course on domestic violence and child abuse, which are serious problems also in Estonia [20], is included in the postgraduate residence training curriculum.

Concerning the family doctors' attitudes to the importance of awareness of their patients' family-related issues, the results indicated that the awareness of drug addiction, diseases of family members, living conditions of the family and alcohol problems in the family were considered the most essential. The awareness of the patients' relationship problems, economic problems and leisure activities was not so highly valued. A recent survey among patients in Estonia revealed that they were also more disposed to involve the family physician in such problems as harmful habits and diseases in the family, but they were less willing to share the relationship problems [15]. This can reflect the current situation in Estonia where the number of drug users as well as alcohol users has significantly increased during the last five years [21]. Lately, much attention has been paid to the problem by politicians, doctors and the mass media.

The low willingness to be aware of the patients' relationship problems is partly related to insufficient preparedness in this field, as the doctors who considered themselves to be adequately prepared to tackle relationship issues were also more often willing to do this. From another point of view, family issues, especially relationships and economic situation, are always delicate topics and require consideration of the patient's attitude to corresponding activities. It has been shown that patients vary considerably in their preferences for physician inquiries into such problems as social functioning, psychosocial issues and health risks. Also, it may reflect cultural differences: in the Nordic countries biomedical talk is more common, while in the southern regions psychosocial dialogue is prevalent [22].

Although most of the family doctors agreed that modifying of the health damaging risk factors (smoking, alcohol and drug abuse) of their patients was their task, they also felt that management of these problems was ineffective, or they perceived themselves as poorly prepared, or had lack of time for such activities. This shows that despite the fact that family doctors are becoming increasingly more aware of their role, there exists the actual need to improve their instruments for handling lifestyle related and psychosocial problems. In practice, both individual and family–centered working methods are needed, while the choice depends on the patient's problems and needs [23]. Management of such issues requires development of new interviewing strategies and different ways to use the visit time more effectively [24]. Finnish experience shows that after completing an education programme, the family doctors' became more family-oriented and family doctors satisfaction with their work was also increased [25].

## Conclusions

The results of the present study allow to conclude that Estonian family practitioners are favourably disposed to involvement in family-related problems, but they need additional training especially in the field of relationship management.

## Competing interests

The author(s) declare that they have no competing interests.

## Authors' contributions

All authors participated in the design of the study. MO and RK carried out the data collection, performed the data analyses and drafted the manuscript. All authors participated in the discussion of the drafts. All authors read and approved the final manuscript.

## Pre-publication history

The pre-publication history for this paper can be accessed here:


